# The Effect of Environmental Performance on Employment: Evidence from China’s Manufacturing Industries

**DOI:** 10.3390/ijerph16122232

**Published:** 2019-06-25

**Authors:** Wei Shan, Jingyi Wang

**Affiliations:** 1 School of Economics and Management, Beihang University, Beijing 100191, China; amanda1249@126.com; 2 Key Laboratory of Complex System Analysis and Management Decision, Ministry of Education, Beijing 100191, China

**Keywords:** environmental performance, environmental regulation, green innovation, employment, panel vector autoregressive model

## Abstract

This research aims to explore the interaction between environmental performance and employment China’s manufacturing industries. Based on the environmental performance of 32 industries in China’s manufacturing industry during 2006–2015, a panel vector autoregressive model was constructed to study the interaction between industry output and employment in clean industries and dirty industries. The dynamic impact and internal transmission mechanism between environmental performance is analyzed. The study found that in the early stage, due to the reduction of production scale, there was a weak and short-term negative correlation effect on employment, and the mutual promotion relationship between economic benefits and employment was unsustainable. In return, employment affects environmental performance, but the effect differs due to the different forms of environmental performance. For dirty industries, the impact of environmental performance on employment through technical effects is more significant and, thus, a win–win situation of ecological environment and employment stability will be achieved. This research has practical significance regarding how to scientifically and effectively carry out environmental regulation and green management.

## 1. Introduction

Achieving sustainable growth and improving people’s livelihood have always been the two major concerns of the Chinese government. The current mode of economic development in China is typified by high growth, energy consumption, and pollution characteristics, and this has caused great stress on both energy consumption and the environment [1]. Environmental issues and employment issues bear the brunt. Since the new century, China’s industrialization process has accelerated and has shown the basic characteristics of the post-industrial era. The manufacturing industry is at the industrial center and at the core of maintaining stable economic development. However, development of the manufacturing industry inevitably brings about deterioration of the environment. Focusing on solving outstanding environmental problems, prevention and control of the source, and environmental pollution such as air pollution, water pollution, soil pollution, and solid waste pollution have become increasingly prominent in social and economic development. In turn, the country’s industrial structure and employment situation are affected. Since the 1990s, China’s employment elasticity has remained at a low level, and the employment situation has become increasingly severe. In 2013, the pollution level of almost all industries of recent decades reached their peak, and this also aroused the government’s high concern. Under the guidance of the dual national policy of environmental protection and energy conservation, the problems of resource waste and pollution emissions in dirty industries and the phenomenon of breaking the ecological balance have been greatly alleviated.

Based on this, this paper intends to explore the “win–win” approach to sustainable development and people’s livelihood from the perspective of China’s manufacturing industry, from the three dimensions of environment, economy, and employment. Taking the interaction between environmental performance and employment in China’s manufacturing industry as the starting point of research, based on the industry’s perspective, we aim to explore how to conduct environmental management scientifically and effectively, and propose policy opinions and suggestions according to the differences of the industry. Through a combination of quantitative and qualitative research, a dynamic model is built to simulate the long- and short-term effects of environmental performance and employment in both clean and dirty industries, to deepen the understanding of this interactive shock response process.

Regarding whether sustainability and economic benefits can be mutually beneficial, domestic and foreign scholars are in disagreement. At the beginning, the mainstream academic circles were restrained, that environmental regulation inhibited the growth of economic benefits by increasing the production cost of enterprises and the price of labor factors [2]. Some researchers verified similar conclusions through the choice of companies in the chemical, petroleum, pulp and paper, and furniture manufacturing industries [3,4]. In addition to reducing the economic output by reducing industrial output, environmental regulation also has a negative impact on economic efficiency through the decline of industrial productivity. Barber and Mc Connell used the US chemical, paper, and other polluting industries as research objects, and found that the increase in investment in environmental governance during 1960–1980 led to a decline in industrial productivity, with the largest decline reaching nearly 30% [5]. Dufour et al., Gollop and Robert, Gray and Shadbegian, Boyd and Mc Clelland have reached similar conclusions in their research into the power, paper, petroleum, and steel industries [6,7,8,9]. Conversely, some scholars support environmental performance to promote economic growth by improving output or increasing productivity. Boyd and Mc Clelland pointed out that environmental regulation may lead to a reduction in potential output but, at the same time, there may be a coexistence of increased output and reduced pollution, thereby achieving a win–win situation for environmental protection and economic growth [10,11,12]. Hamamoto confirmed it through research on company’s technological innovation activities [13]. It is recommended that companies combine cleaner production and environmental management to increase the results of sustainable innovation and financial gain [14]. However, such results do not apply to all regions. Martin et al. found that the impact of the implementation of the United Kingdom’s energy tax on manufacturing output was not significant. Environmental productivity and corporate efficiency are found greater for export-oriented firms [15]. Brannlund et al. found that environmental regulation was negatively correlated with the productivity of some enterprises, but that some companies’ productivity was not significantly related to environmental regulation [16]. Lanoie, Lajeunesse and Patry found that there was a lag in the impact of environmental performance on firm productivity [17], where technical change was the main driver of most total factor productivity growth [18]. Due to the introduction of the North American Free Trade Agreement (NAFTA), Grossman and Krueger introduced the relationship between environmental and economic growth with an inverted “U” curve [19]. Shafik and Bandyopadya also found that the indicators of per capita income and air pollutants also showed an inverted “U” relationship [20]. This conclusion has a certain similarity with Kuznets’ proposed Kuznets curve, so the above relationship can also be called the environmental Kuznets curve [21]. A specific characteristic of the curve is that the environmental quality will show an inverted U-shaped trend with economic growth, that is, the environmental quality will gradually deteriorate and then gradually improve [22,23]. Domestic scholars have taken the data of various industries in China as the research object and also discover the differences, interactivity, lag and complexity of environmental regulation and the industrial growth impact mechanism [24,25,26,27,28].

Following the rise of the so-called “green economy” paradigm, research has mainly focused on the relationship between the rise of the green economy and the effects on creation of new opportunities of green employment [29]. So, can environmental performance drive employment growth? Summarizing the existing viewpoints, the employment effects of environmental regulation can be roughly classified into three types: scale effect, substitution effect, and the uncertainty effect brought by technological innovation [30]. The scale effect, that is, environmental regulation, places enterprises in an unfavorable competitive position in the world by increasing the production cost of enterprises and increasing the cost of pollution control and, thus, the labor demand is reduced. The early research is more inclined to take this view. Henderson and Greenstone believe that environmental regulation will lead to a reduction in production scale due to the increase in production costs which, in turn, will lead to a decrease in employment in the industry, that is, environmental regulation will have a negative effect on employment in the industry [31,32]. The Environmental Pollution Costs and Expenses Report (PACE), written by the US Census Bureau in 1993, also validates this conclusion. The substitution effect, that is, environmental regulation, makes enterprises more inclined to use labor factors instead of price-enhancing resource-type elements due to the increased price of resource-based elements. Bezdek, Shimer et al. verified this conclusion [33,34,35,36]. According to the World Labor Report published in 2009, moderate environmental regulation can achieve the two goals of improving environmental conditions and stimulating labor demand, and achieving the dual dividend of ecological environment and employment stability. It is also called the “double dividend hypothesis”. Chen also found that environmental performance achieved employment growth through the substitution relationship between pollutant elements and labor factors [37,38,39,40,41].

The impact of environmental change on employment, as triggered by technological change, has greater uncertainty. The first is promotion theory, including the typical “porter hypothesis” and “double dividend” [42]. Pianta and Vivarelli conducted statistical analysis on 21 departments in 5 countries, including Italy, Finland, Norway, Germany, and Denmark. The innovation activities of enterprises have a positive effect on overall employment. Innovation will lead to employment decline in a short time, but only within the department [43]. According to Krugman and Young, the increase in labor factors and capital factor inputs will not continue to stimulate economic growth, and sustainable economic growth comes from continuous innovation [44]. Material productivity improvements are found to receive targeted public financial support for realizing eco-innovations [45]. The second is suppression theory, where it is believed that environmental governance will lead to the emergence of new sectors, which will lead to the gradual decline of the traditional sector, and thus lead to structural unemployment [46,47]. The third is comprehensive theory. At different stages of development, the effects of different strengths and different purposes of environmental regulation on employment in different industries and industries of different scales are also very complicated. First, the impact mechanisms of different industries are different [48]. Some scholars have also found that in the process of green energy, environmental regulation has a negative impact on employment in manufacturing industries with high energy consumption, and has a positive impact on employment in the electricity, gas and water production, and supply industries [49,50,51]. From a macro perspective, the impact of environmental regulation on employment is not only reflected in the number of jobs but, in many cases, it will show changes in the distribution of employment in different industries, and there are significant differences between different industries. There are also some scholars who analyze the problem from the perspective of the structure of the employed [3,52]. Since environmental regulation has always been guided by green innovation, its development orientation has a green and sustainable basis, that is, employment or industry meets the requirements of low carbon emissions, energy conservation, environmental protection, and pollution reduction. Taking China’s manufacturing industry as an example, the formulation and implementation of environmental regulation has greatly promoted the development of a typical “green industry” in the comprehensive utilization of waste resources. A similar law of labor mobility will also occur as an interdepartmental microenterprise. At different stages of development, the comprehensive impact mechanism of environmental regulation on employment will also change, and some scholars have verified the U-shaped relationship between the two [53,54].

In summary, the correlation between environmental performance and employment differ according to the existing research. Foreign studies mainly focus on the needs of enterprises in developed countries, and the research is increasingly theoretical, detailed, and empirical, while the domestic relationship between environmental regulation and employment in the industry is rarely mentioned. There are few studies and the subjects are relatively one-sided and, in empirical research, the main focus is on static analysis methods. This paper takes the development of environmental regulation and employment in China’s manufacturing industry as the starting point of the research, based on the perspective of industry, and aims to explore how to carry out environmental regulation scientifically and effectively. Through the combination of quantitative and qualitative research, we explore the dynamic impact response process of the environmental performance of the clean industries and the dirty industries on employment, and deepen understanding of the interaction between the two. Strengthening the depth and breadth of theoretical research has great practical significance for how to scientifically and effectively carry out environmental performance and green management.

## 2. Materials and Methods 

### 2.1. Econometric Model

In order to analyze the interaction between environmental performance and manufacturing employment, and to describe the dynamic impact response mechanism between the two, this paper intends to use the panel vector autoregressive model to measure the environmental performance, industry scale economy, and industry employment structure of China’s manufacturing industry during the decade of 2006–2015. The industry panel data consisting of three dimensions is modeled.

The vector autoregressive model (VAR model) can be regarded as a vector autoregressive model consisting of multivariate time series derived from a univariate autoregressive model to estimate the joint dynamic relationship between endogenous variables in the economic system. It explains the dynamic impact of economic shocks on the various endogenous variables in the system without having to assume constraints. The panel vector autoregressive model (PVAR model) was first proposed by Holtz, which introduced panel data based on the autoregressive model. It allows individual effects and heteroscedasticity in the data because there are a large number of cross-sectional units that allow the hysteresis coefficients to change over time, thereby relaxing the time-smooth assumption of the data, and without needing to satisfy general conditions. The PVAR model inherits the advantages of the normal VAR model without distinguishing whether the variables in the model are exogenous or endogenous. Instead, all variables are treated as endogenous variables and the relationships between variables can actually be compared. By orthogonalizing the impulse response function, the influence of endogenous variables on other endogenous variables can be separated so that the degree of influence of changes in one variable on other variables can be analyzed; the PVAR model allows individual effects and time effects, and individual effects allow for different observations and individual differences between units. The time effect reflects the common effects that different observation units may have on the cross section. Therefore, the PVAR model is more widely used in studying econometric problems. The construction process of the PVAR model is as follows:

(1) $$y_{it}=\alpha_{0t}+\sum_{l=1}^{m}\alpha_{lt}y_{it-l}+\sum_{l=1}^{m}\delta_{lt}x_{it-l}+\varphi_{t}f_{i}+\mu_{it}$$

*i* = 1, 2, …, *N*; *t* = 1, 2, …, *T*.

In Formula (1), *N* represents the number of individuals observable in the sample, *T* represents the length of the interval of the time series of the sample data, *i* represents the individual in the sample, *t* represents the time, and $y_{it}$ represents the *m* × 1 vector in which *m* observable random variables of the individual *i* at time *t*. $x_{it}$ represents the *m* × 1 vector of the observable deterministic strict exogenous variable of the individual *i* at time *t*, and *f_i_* represents the individual effect vector of the unobservable individual *i*, $\alpha_{0t}$,$\;\alpha_{lt}$, $\delta_{lt},\;\psi_{t}$, respectively represent the coefficient vector of the equation regression, $\mu_{it}$ is the model error term.

### 2.2. Variables and Data Sources

Environmental performance indicators that have been frequently used in research include pollutant emissions, pollutant capital inputs, and energy consumption. Some factors will also be measured in terms of public environmental awareness and public environmental preferences [55,56,57,58], since public perceptions of the environment are becoming core elements in promoting environmental sustainability [59]. To systematically measure the intensity of environmental governance, Dam and Scholtens conduct comprehensive assessments on four levels: environmental management, environmental policy, environmental improvement, and environmental performance and impact. Environmental input indicators include inputs based on environmental innovation and inputs based on pollution control, and environmental inputs are measured using environmental R&D budgets, environmental R&D investment, environmental R&D personnel, and other related intangible assets. From the perspective of environmental performance, Ghisetti and Rennings measure the environment of the enterprise by using nine indicators, such as raw material consumption per unit output, energy consumption per unit of output, CO_2_ emissions, air pollution, water pollution, soil pollution, noise pollution, performances, and the ratio of pollutant emissions to total industry output value. SO_2_ removal rate, wastewater compliance rate, solid waste comprehensive utilization, and other indicators were used to measure the intensity of environmental regulation [60,61,62]. Ntanos S etc. examined the relationship between energy consumption and countries’ economic growth or citizens’ life quality in European countries [63,64].

Through the existing research results, research purposes, and data availability, the endogenous variables selected in the PVAR model include industry employment, industry development, and industry environmental performance. The number of employed people in the industry measures the employment situation of the industry; the total industrial output value measures the development of the industry, reflecting the total scale and total level of industrial production in a certain period of time, and also reflects the economic performance of the industry to a certain extent, and is a key factor in promoting employment. The variable indicators and data sources involved in the model are shown in Table 1. In order to eliminate the impact of inflation, the data processing of the industry’s output value is based on 2006, and the data is adjusted through the consumer price index CPI. In order to eliminate the influence of heteroscedasticity, the industry’s employment and industry’s total output value are taken from a natural logarithm describing the growth rate of the industry and the growth rate of the industry’s output value. Since the collection of statistics regarding industrial pollutants processing capacity in China Environmental Statistical Yearbook began in 2006, the data sources are mainly based on panel data in the past ten years since 2006.

Dangelico and Pujari found that the degree of environmental pollution in enterprises varies, and the impact of green innovation on the economic performance of enterprises also varies [65]. Kunapatarawonga and Martinez-Ros have also reached similar conclusions in their research. It is found that the behavior of enterprises in dirty industries is more likely to be of concern to the public, so the adoption of green innovation activities will create greater value for enterprises in dirty industries. In comparison, green innovation in clean industries has received less attention from the public [66]. Based on the existing literature, this paper classifies clean industries and dirty industries according to the toxic emission index of each industry in the 2015 US Toxic Substances Emission Inventory Report as shown in Table 2. The average employment scale and average sales value of clean industries and dirty industries are similar, and the main difference is reflected in energy consumption, pollutant emissions, and treatment volume. For dirty industries, the wastewater treatment capacity is significantly higher than that of clean industries, but the emphasis on waste gas and solid waste is significantly less than that of wastewater treatment. The trends of the indicators in the ten years are basically the same, and the indicators in clean industries are more stable. It is worth noting that in 2013, the average wastewater discharge of the industry produced large fluctuations, and the degree of change in dirty industries was more obvious. The wastewater discharge of the two industries even reached ten times of the same period, but based on an indicator from 2014. It has since fallen sharply, and it can be speculated that this has a lot to do with the high-intensity environmental regulations at the time. In addition, the average increase in the number of exhaust facilities and waste gas utilization capacity in both types of industries is higher than that in wastewater treatment and solid waste treatment. Industrial waste gas is an important source of air pollution and environmental pollution, and it is also one of the important causes of haze weather in most parts of China. Therefore, waste gas utilization has become the focus of the government and has been closely monitored by the public. Under the constraints of high-intensity environmental regulations, the investment in enterprise waste gas utilization equipment has increased, the treatment capacity of exhaust gas has increased, and the growth rate of exhaust gas emissions has slowed down.

According to the results of environmental pollution and environmental governance-related indicators in the clean industries and the dirty industries, as shown in Table 3, the average employment scale of the clean industries and the dirty industries is similar, the industrial pollution is slightly lower than the average sales value, and the overall industry pollution is slightly lower. Energy consumption and pollutant emissions and treatment volume are significantly higher than the clean industries. However, the importance of waste gas and solid waste is less than that of wastewater treatment, which is slightly lower than or even significantly lower than that of the clean industries. This conclusion is reflected in the selection and analysis of model variables in the following.

## 3. Results

### 3.1. Interaction between Environmental Performance and Employment in Clean Industries

#### 3.1.1. Variable Stationarity Test

Assuring the stability of the time series in the system is a prerequisite for constructing the vector autoregressive model and performing the Granger causality test. If the time series is not stable, the cointegration test should be performed on the basis of the first-order or second-order differential stationary. In this paper, the test methods of various panel unit roots, such as ADF, LLC, and PP, are used to check the stability of the panel data. Each variable passes the stationarity test (wastewater treatment capacity wwr is not considered in the optimized model), as Table 4 shows.

#### 3.1.2. Results in Clean Industries

The PVAR model of each economic variable was constructed by using Stata12.0 (StataCorp, College Station, TX, USA). The analysis results are shown in Table 4, in which the statistic adjusted by white heteroscedasticity is indicated in parentheses, and the first column represents the variable of lag phase. It can be seen from the estimation results in Table 5 that employment and output value have a positive impact on employment, and waste gas utilization capacity has a negative impact on employment; waste disposal capacity has a positive impact on itself, and employment and energy consumption have a negative impact; the impact of employment on output value is positive, and the impact of waste gas utilization capacity on output value is negative.

Similar to the problems set by other econometric models, the determination of the lag order p in the vector autoregressive model has certain subjective factors. Through the weighted processing of the log-likelihood function value and the number of parameters, the trade-off criteria of the commonly used three measurement models include the AIC criterion, the BIC criterion, and the HQIC criterion, as the criterion for determining the lag order of the vector autoregressive model. Combining the trade-off results of the above three criteria, p = 1 in the model, that is, the first-order lag variable is the optimal instrumental variable.

#### 3.1.3. Correlationship between Environmental Performance and Employment in Clean Industries

**Figure 1:**
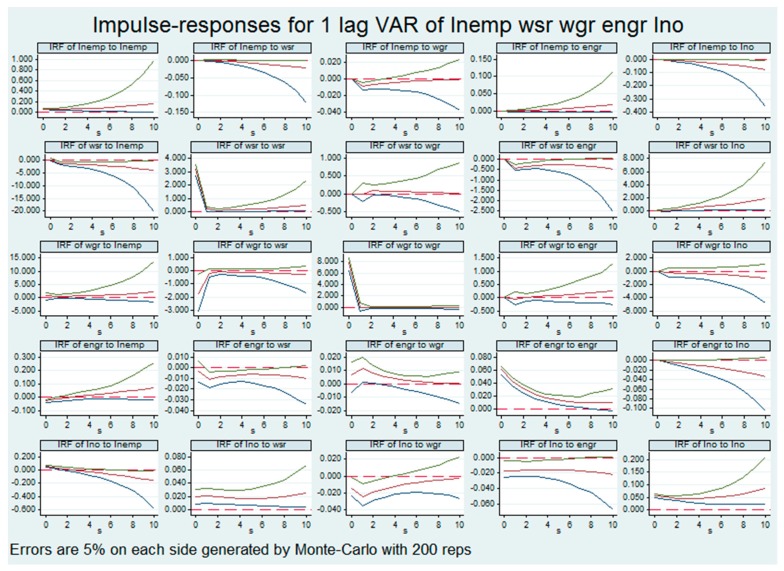
Clean industries impulse response function.

The impulse response function (IRF) is used to analyze the output (response) of the system when the input is a unit impulse function, which solves the problem that the interpretation of the univariate parameter estimation value is difficult in the panel vector autoregressive model. The impulse response function investigates the response of the current and future values of the system’s endogenous variables when the disturbance term is 0 when the other endogenous variables are subjected to a random shock, that is, the economic variables of the system when the variable is influenced. The impulse response function of each variable in the PVAR model in clean industries is shown in Figure 1.

The first row in Figure 1 shows the impact of employment in clean industries on itself and other indicators. The number of employed people had a positive response to their own impact, and this positive shock response gradually increased over time. From the perspective of the impact response of the number of employed people on the capacity of waste disposal, the impact of the number of employed persons on the capacity of waste treatment was 0 in the initial stage, and a negative reaction occurred near the third stage, and this negative effect gradually increased over time. That is, in clean industries, the more people employed, the smaller the proportion of solid waste disposal. On the one hand, the growth of the industry led to the growth of the scale of the industry, which led to an increase in the amount of solid waste generated. On the other hand, in clean industries, the amount of solid waste generated was much smaller than that of dirty industries. The treatment did not receive attention. Correspondingly, the impact of the first three employments on waste gas utilization capacity was also negative, but the effect was not significant after the third period. The positive impact of employment on energy consumption and the negative impact on output value also gradually increased.

The first column shows the impact of each variable on the number of people employed. The waste disposal capacity will have a weak positive impact on employment at the beginning of the period, but will change to a negative one over one period. Energy consumption has a weak negative effect on employment at the beginning of the period but, after the first period, turned into a positive impact, with the impact becoming stronger. The impact of industry output on employment also shifted from positive to negative.

#### 3.1.4. Granger Causality Test

On this basis, the Granger causality test, as shown in Table 6, shows that employment itself and industry output are factors that affect employment. Employment and energy consumption can explain changes in waste handling capacity. The treatment capacity of waste gas and waste can explain changes in energy consumption, and employment and waste gas utilization capacity can explain changes in production.

The scale, technical, and structural effects provide a theoretical basis for analyzing the relationship between environmental performances and manufacturing employment. (1) Scale effect. The Austrian affirmation law proposed by the American economist Arthur Oken describes the quantitative law of the stability between GDP and unemployment rate, and demonstrates the positive correlation between economic growth and employment. This law recognizes that for every 1% increase in production, the number of employed people has risen by less than 1%. The reason may be that the increase in production is achieved by increasing the workload of workers, not by increasing the number of employed people, but by increasing the number of second occupations, thereby making the employment increase less than the percentage increase in production. This theory is similar to the conclusion of economic growth theory. Economic growth is the result of an increase in social wealth caused by factors such as an increase in the input of production factors or an increase in the efficiency of factor production in a country or region. According to these two classical economic theories, the economic growth and scale expansion of the industry are accompanied by the increase of labor factors, and also generate a large amount of pollution emissions in the production process. Therefore, the increase in employment will be negatively correlated with the environmental governance capacity. Under the influence of environmental performance, the investment in environmental governance is enhanced, the production cost of the industry is increased, and the competitive advantage of the industry is weakened. As a result, the industry reduces production and the scale of employment decreases. (2) Technical effects. The growth of employment in the industry will drive the growth of the industry scale and the improvement of economic interests. Then, more funds or more production factors will be invested in the research and development of environmental governance and clean energy research, thereby further promoting the competitive advantage of the industry while achieving environmental governance capacity and production efficiency has improved, and employment has a positive correlation with environmental governance capabilities. On the contrary, the improvement of environmental performance level promotes the innovation and development of the industry while bringing fresh blood to the industry. (3) Structural effects. At present, the pollution management of the manufacturing industry is still in the development stage. With the economic growth of the industry, the factor input structure and output structure of the industry change, and the production mode and development mode are changing from the traditional extensive production mode to the lean production mode. In this development stage, the industry is from the environment. Both the performance perspective and the market-oriented perspective are shifting towards sustainable development, and the intensity of labor will change during this period.

For clean industries, the optimized model includes waste gas utilization capacity, waste treatment capacity and energy consumption indicators, and the waste gas utilization capacity affects the scale effect of total employment by changing the industry output at the beginning. The treatment capacity of pollutants will have a negative effect on total employment by affecting the total output. That is, the treatment of pollutants will increase the production cost of enterprises and reduce the output of enterprises, and ultimately reduce employment. However, this effect will be weakened after the first phase. On the basis of the above, the energy consumption index is further introduced, that is, the environmental performance affects the employment substitution effect by influencing factor input. The experimental results analyze the positive substitution effect of environmental performance on employment, but the experimental results show that the environmental performance implementation effect Among them, the stronger the waste gas utilization capacity, the higher the energy consumption, indicating that the process of treating waste gas in clean industries is accompanied by energy consumption. At the same time, energy consumption and waste disposal capacity are negatively correlated, that is, low energy consumption and high waste solid processing capacity can be achieved in clean industries. On the one hand, the reduction of energy consumption has eliminated some enterprises that rely on traditional energy sources and, on the other hand, promoted the technological innovation of the industry and the use of clean energy, thereby reducing the generation of solid waste and, thus, improving the waste treatment capacity. Conversely, the improvement of waste treatment capacity may also be caused by technological effects or scale effects, that is, the reduction of pollution control technology or the reduction of waste emissions caused by the exit of highly polluting enterprises while achieving a reduction in energy consumption. The relationship between changes in energy consumption and employment is not significant, but employment has a negative impact on both production and waste disposal capacity.

### 3.2. Interaction between Environmental Performance and Employment in Dirty Industries

#### 3.2.1. Variable Stationarity Test

The variables in the pollution industry model are I(1) sequences. As shown in Table 7, the cointegration test needs to be further performed on the basis of first-order differential stationary. As shown in Table 8, the PVAR model of dirty industries rejects the null hypothesis by the co-integration test, that is, there is a cointegration relationship between variables. Therefore, the first-order difference is constructed on the variable to construct the PVAR model.

#### 3.2.2. Interaction Results between Environmental Performance and Employment in Dirty Industries

It can be seen from the estimation results in Table 9 that, unlike clean industries, the correlation between waste gas utilization capacity and energy consumption and other variables in dirty industries is weak. Among them, employment is still mainly affected by the positive impact of the previous employment and the previous period of output, and the output value and employment are still mutually reinforcing; the impact of employment on the treatment capacity of wastewater and waste is negative, but the wastewater treatment capacity and the impact on employment are positive, and the impact of waste disposal capacity on employment is negative while the impact of output value on waste disposal capacity is positive.

#### 3.2.3. Correlationship between Environmental Performance and Employment in Dirty Industries

**Figure 2:**
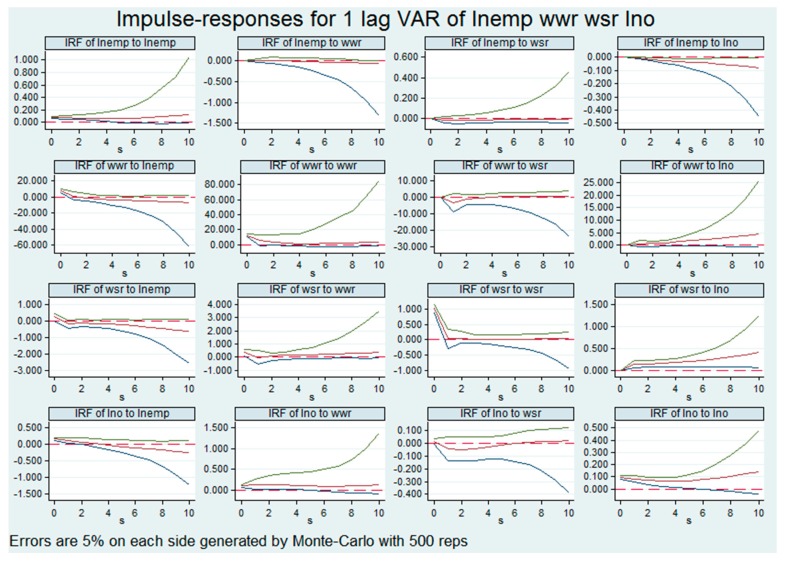
Clean industries impulse response function.

The impulse response function is also used to analyze the PVAR model of dirty industries. In Figure 2, the number of employed people has a positive response to their own impact, and this positive impact response gradually increases with time. From the perspective of the impact response of employment on the capacity of waste disposal, the impact of the number of employed people on the capacity of waste disposal has a weak negative reaction in the vicinity of the first three periods. This negative effect gradually changes over time. The impact on wastewater treatment capacity also has a relatively weak negative impact. The impact of employment on output value is the same as that of clean industries. The initial stage is in a positive correlation, but its negative impact has gradually increased over time, but the relationship is positive between employment in dirty industries and the initial stage of economic growth and is weaker than clean industries. The wastewater treatment capacity and waste treatment capacity are generally consistent with the impact of waste treatment capacity in clean industries. At the beginning of the period, there will be a weak positive impact on employment, but from the first period to the negative impact. The impact of industry output on employment is also shifting from positive to negative.

#### 3.2.4. Granger Causality Test

The Granger test results are shown in Table 10. The test results show that employment itself and industry output in dirty industries are factors affecting employment. Employment can explain changes in wastewater and waste treatment capacity. Industry output can explain changes in waste treatment capacity, wastewater treatment. Capacity and employment can explain changes in production. The effects between the remaining variables did not pass the Granger causality test.

The optimized model in dirty industries, the indicators of environmental performance include wastewater treatment capacity and waste treatment capacity, and energy consumption and waste gas utilization capacity no longer have a negative correlation, and the impact with other variables is not significant. Among them, the impact of industry output value and pollutant treatment capacity is more significant, especially in wastewater treatment. Compared with clean industries, the positive correlation between sales value and employment in dirty industries is shorter, and the impact is relatively smaller. However, the growth of employment still led to a small decrease in wastewater discharge capacity and solid waste utilization rate. On the one hand, it is caused by the increase in emissions caused by scale effect. The pollution industry not only brings about more pollution emissions from the production process, but also expands. The scale and increased output inevitably have a negative impact on the ecological environment. On the other hand, it also reflects the lagging effect of response measures under environmental performance. However, the duration of this weak negative effect is not long, and the specific reasons are also traced back to the dynamic impact between employment and output. On the contrary, the capacity of wastewater treatment has driven the employment growth of the industry. On the one hand, it is attributed to the change of employment structure, that is, the resource input of wastewater treatment has contributed more jobs, and on the other hand, it has benefited from the transformation of dirty industries. In 2013, pollution various industries had reached their peak over several decades, which also aroused great attention from the government. Especially for dirty industries with large energy consumption, with great ecological damage and causing extreme shortage of resources, the environmental performance constraint has been greatly enhanced. Under the guidance of the dual national policy of environmental protection and energy conservation, the waste of resources, pollution discharge, and breaking of ecological balance in dirty industries have been greatly alleviated. In 2014, environmental management work achieved remarkable results. According to Porter’s hypothesis, moderate environmental performance is beneficial for stimulating technological innovation, optimizing resource allocation, improving resource utilization, offsetting the increase in production costs brought about by environmental performance, improving industry competitiveness, and reducing employment caused by economies of scale. At the same time, whether from the perspective of the government or the public, social recognition through pollution control or sustainable green products will bring industry scale to the enterprise, and will also benefit from the introduction of new technology or new products and gain a first mover advantage. This mechanism is reflected in the increase in the scale of the industry and the improvement in economic efficiency, while increasing the employment capacity of the solid waste.

## 4. Discussion

This paper selects the environmental performance indicators of 32 industries in China’s manufacturing industry in 2006–2015, and constructs a panel vector autoregressive model to study the interaction between environmental performance, industry scale, and industry employment. The following conclusions are drawn.

For clean industries, the waste gas utilization capacity affects the scale effect of total employment by changing the industrial output, but this effect will be weakened, and the waste gas utilization capacity will positively affect employment. The impact will increase over time, and the scale effect will be offset, possibly due to labor mobility or technical effects of the second occupation resulting from waste gas utilization, since there is an inverse relationship between firm productivity and pollution emissions [67]. At the same time, low energy consumption and high waste solid treatment capacity can be achieved in clean industries. On the other hand, the stronger the waste gas utilization capacity, the higher the energy consumption, indicating that the waste gas utilization process in clean industries is accompanied by energy consumption. Employment has a negative impact on the ability to dispose of waste. 

For dirty industries, the impact of energy consumption and other variables is no longer significant. The impact of industry output value and pollutant treatment capacity is more significant, especially in wastewater treatment. Compared with clean industries, the positive correlation between sales value and employment in dirty industries is shorter and the impact is relatively smaller. However, the growth of employment still leads to a small decrease in wastewater discharge capacity and solid waste utilization rate, which is the result of the combination of scale effect and environmental performance. On the contrary, the ability of wastewater treatment has driven the employment growth of the industry. On the one hand, it is attributed to the change of employment structure and, on the other hand, it has also benefited from the transformation of dirty industries.

Since this paper is based on the industry perspective, the selected data indicators are limited to the relevant data of the manufacturing industry. The existing statistical data are limited, and the years of the available indicators are limited. Therefore, the limitation of the sample size in the study may affect the reliability and validity of the model. On the other hand, the conclusions of the cointegration and Granger causality tests are only a statistical estimate and not a true causal relationship, and cannot be used as a basis for affirming or negating causality. Therefore, in future research, investigation of the economic principles behind the research results is still necessary.

## 5. Conclusions

Pursuing environmentally friendly (green) performance poses several challenges, but it also affords opportunities to create new methodologies for generating a competitive advantage for manufacturing companies [68]. For enterprises, moderate environmental regulation is conducive to stimulating technological innovation and optimizing resource allocation. Therefore, improving resource utilization can offset the increase in production costs brought about by environmental performance, improving industry competitiveness and reducing employment caused by economies of scale. They will also benefit from the introduction of new technology or new products through the first mover advantage. At the same time, whether from the perspective of the government or the public, social recognition through pollution control or the sustainable green products will bring industry scale to the enterprise, and this mechanism is reflected in the increase in the scale of the industry and the improvement in economic efficiency, while increasing the employment capacity of the solid waste. This is especially evident in dirty industries. Furthermore, it is necessary for the government to adopt different policy instruments for different industries.

## Figures and Tables

**Table 1:** Variable meanings and data sources.

Variable	Meanings	Calculation	Data Sources
Lnemp	Employment	ln (average number of employees)	China Industrial Economics Statistical Yearbook
Wwr	Wastewater utilization capacity	Industrial wastewater treated/industrial wastewater produced	China Environmental Statistics Yearbook
Wgr	Waste gas utilization capacity	Industrial waste gas treated/industrial waste gas emission	China Environmental Statistics Yearbook
Wsr	Solid wastes utilization capacity	Industrial solid wastes utilized/industrial solid wastes produced	China Environmental Statistics Yearbook
Engr	Energy consumption	Total energy consumption/total industry output	China Energy Statistics Yearbook
Lno	output value	ln (industrial sales value of industrial enterprises above designated size)	China Statistical Yearbook

**Table 2:** Composition of industry categories.

Clean Industries	Dirty Industries
Agricultural and sideline food processing	Coal mining and dressing
Food production	Extraction of petroleum and natural gas
Beverage production	Ferrous metal mining and dressing
Tobacco products processing	Non-ferrous metal mining and dressing
Textile industry	Non-metallic mining and dressing
Clothes, shoes, and hat manufacture	Papermaking and paper products
Leather, furs, down, and related products	Petroleum processing, coking, and nuclear fuel processing
Timber processing, bamboo, cane, palm fiber, and straw products	Raw chemical material and chemical products
Furniture manufacturing	Medical and pharmaceutical products
Printing and record medium reproduction	Chemical fiber
Production of cultural, educational, and sports articles	Rubber and plastic products
Metal products	Non-metal mineral products
Ordinary equipment and manufacturing	Smelting & pressing of ferrous metals
Transportation equipment and manufacturing	Smelting & pressing of non-ferrous metals
Electrical machines and apparatuses manufacturing	
Communication equipment, computers, and other electronic equipment	
Communication equipment, computers, and other electronic equipment	

**Table 3:** Characters of clean industries and dirty industry.

Variables	Clean Industries		Dirty Industries	
	Mean	Standard Deviation	Mean	Standard Deviation
Employees (10 thousand persons)	299.8483	219.058	209.3721	172.034
Sales value (100 million yuan)	21,629.65	20,707.11	21,103.76	19,444.1
Total energy consumption (10,000 tons of standard coal)	1812.981	1655.133	13,346.51	16,987.12
Energy consumption rate	1.007999	0.723606	5.282489	3.4602
Wastewater treatment (10,000 tons)	202.2322	300.265	1365.268	2285.545
Exhaust gas treatment (10,000 cubic meters)	8568.291	20,858.05	50,347.77	10,0627
Waste solid utilization (10,000 tons)	347.7328	617.0931	7484.646	9937.406
Wastewater discharge (10,000 tons)	18,570.92	5989.49	10,6875.5	28,572.9
Exhaust emissions (100 million cubic meters)	1985.268	1965.356	23524.53	42,425.11
Waste solid production (10,000 tons)	350.0272	481.3707	13234.69	17134.15
Wastewater treatment capacity	5.18507	2.248337	18.17803	22.55671
Exhaust gas treatment capacity	6.471434	25.16006	2.11569	2.609213
Waste treatment capacity	8.933648	5.838289	7.167836	2.767392

**Table 4:** Stationarity test of the unit root of clean industries panel.

	**lnemp**		**Lno**		**Engr**	
Test	Statistic	Prob.	Statistic	Prob.	Statistic	Prob.
LLC	−12.2744	0.0000	−3.87449	0.0001	−9.5785	0.0000
IPS	−2.71873	0.0033	−1.8623	0.0313	−3.59651	0.0002
ADF	48.9552	0.0434	49.7375	0.0535	76.0270	0.0001
PP	49.2210	0.0499	50.1879	0.0584	85.7209	0.0000
	**wgr**		**Wsr**		**Wwr**	
Test	Statistic	Prob.	Statistic	Prob.	Statistic	Prob.
LLC	−18.4091	0.0000	−6.2398	0.0000	−3.4232	0.0003
IPS	−7.6812	0.0000	−2.25075	0.0122	−1.02823	0.1519
ADF	109.723	0.0000	61.9884	0.0045	46.4680	0.1135
PP	119.462	0.0000	60.2837	0.0068	47.8243	0.0899

**Table 5:** Panel vector autoregressive model (PVAR) estimation results of clean industries.

	lnemp	Wsr	wgr	engr	Lno
L.h_lnemp	1.085 ***	−23.101 ***	11.107	0.104	0.267 *
	−6.89	(−2.69)	−1.1	−0.88	(−1.80)
L.h_wsr	0	0.050 **	−0.029	−0.002 ***	0
	−0.84	−2.38	(−0.75)	(−8.45)	−0.96
L.h_wgr	0.01 ***	0.007	−0.007	0.01 ***	−0.01 *
	(−3.53)	−0.31	(−0.14)	−2.6	(−1.68)
L.h_engr	−0.02	−6.378 ***	−2.018	0.679 ***	−0.03
	(−0.84)	(−4.40)	(−0.51)	−19.37	(−0.98)
L.h_lno	0.113 **	1.796	−4.509	−0.049	0.880 ***
	(−2.49)	−0.78	(−0.68)	(−1.64)	−17.45

Note: *, **, and *** indicate significant levels of significance at 10%, 5%, and 1%, respectively.

**Table 6:** Granger causality test results of clean industries.

Equation	Excluded	Prob > chi2
h_lnemp	h_wgr	0
h_lnemp	h_lno	0.013
h_wsr	h_lnemp	0.007
h_wsr	h_engr	0
h_engr	h_wgr	0.009
h_engr	h_wsr	0
h_lno	h_lnemp	0.072
h_lno	h_wgr	0.093

**Table 7:** Stationarity test of unit root of pollution industry panel.

	**lnemp**		**lno**		**Engr**	
Test	Statistic	Prob.	Statistic	Prob.	Statistic	Prob.
LLC	−4.36423	0.0000	−4.98564	0.0000	−15.2148	0.0000
ADF	54.2408	0.0021	72.5179	0.0000	165.157	0.0000
PP	53.6103	0.0025	80.6809	0.0000	174.515	0.0000
	**wgr**		**wsr**		**Wwr**	
Test	Statistic	Prob.	Statistic	Prob.	Statistic	Prob.
LLC	−16.3394	0.0000	−9.05134	0.0000	−13.5859	0.0000
ADF	172.148	0.0000	116.498	0.0000	192.619	0.0000
PP	213.212	0.0000	140.529	0.0000	223.009	0.0000

**Table 8:** Kao test of cointegration relationship in pollution industry.

	*t*-Statistic	Prob.
ADF	2.627015	0.0043
Residual variance	0.001944	
HAC variance	0.002002	

**Table 9:** PVAR estimation results of dirty industries.

	h_lnemp	h_wwr	h_wsr	h_lno
L.h_lnemp	1.039 ***	−44.735 *	−4.226 **	0.571 *
	−5.56	(−1.71)	(−2.42)	(−1.63)
L.h_wwr	0.02 *	0.484	−0.016	0.005
	−1.6	−1.25	(−0.52)	−0.84
L.h_wsr	−0.013 *	−3.194	0.018	−0.055
	(−1.13)	(−0.99)	−0.11	(−1.04)
L.h_lno	0.081 **	6.07	1.446 ***	0.848 ***
	(−2.01)	−0.84	−3	−6.91

Note: * means significant effect at 10% confidence level, ** means significant effect at 5% confidence level, *** means significant effect at 1% confidence level.

**Table 10:** Granger causality test results of pollution industry.

Equation	Excluded	Prob > chi2
h_lnemp	h_lno	0.044
h_wwr	h_lnemp	0.088
h_wsr	h_lnemp	0.015
h_wsr	h_lno	0.003
h_lno	h_lnemp	0.093
h_lno	h_wwr	0.091
